# An Unusual Case Report of a Toddler with Metastatic Neuroblastoma Mimicking Myasthenia Gravis

**DOI:** 10.21980/J8G35V

**Published:** 2022-01-15

**Authors:** Raymen Rammy Assaf

**Affiliations:** * Harbor UCLA Medical Center, Department of Pediatric Emergency Medicine, Torrance, CA; ^ Children’s Hospital of Orange County, Emergency Medicine Specialists of Orange County, Orange, CA

## Abstract

Pediatric neuroblastoma is famously characterized by an abdominal mass in a toddler with “raccoon eyes,” Horner syndrome, and/or opsoclonus myoclonus. However, rare cases may present with non-discrete symptoms and signs which create challenges for timely diagnosis. This case depicts a toddler with bulbar symptoms, including *bilateral* ptosis, dysphagia, drooling, head tilt and ataxia in a post-viral course mimicking other neurologic diagnoses, including myasthenia gravis. Magnetic resonance imaging (MRI) of the brain and spine uncovered a right adrenal mass along with heterogenous enhancement in multiple vertebral bodies and the clivus, consistent with metastatic disease. A diagnosis of neuroblastoma was confirmed with elevated homovanillic acid (HMA) and vanillylmandelic acid (VMA) levels and adrenal biopsy. The patient was treated with plasmapheresis for suspected paraneoplastic neurological syndrome and subsequent chemotherapy. In this case presentation, we review neurologic syndromes causing acute-onset pediatric bulbar weakness along with the patient’s key MRI findings.

**Topics:**

Toddler weakness, neuroblastoma, paraneoplastic syndrome.

[Fig f1-jetem-7-1-v26] [Fig f2-jetem-7-1-v26] [Fig f3-jetem-7-1-v26] [Fig f4-jetem-7-1-v26] [Fig f5-jetem-7-1-v26]

**Figure f1-jetem-7-1-v26:**
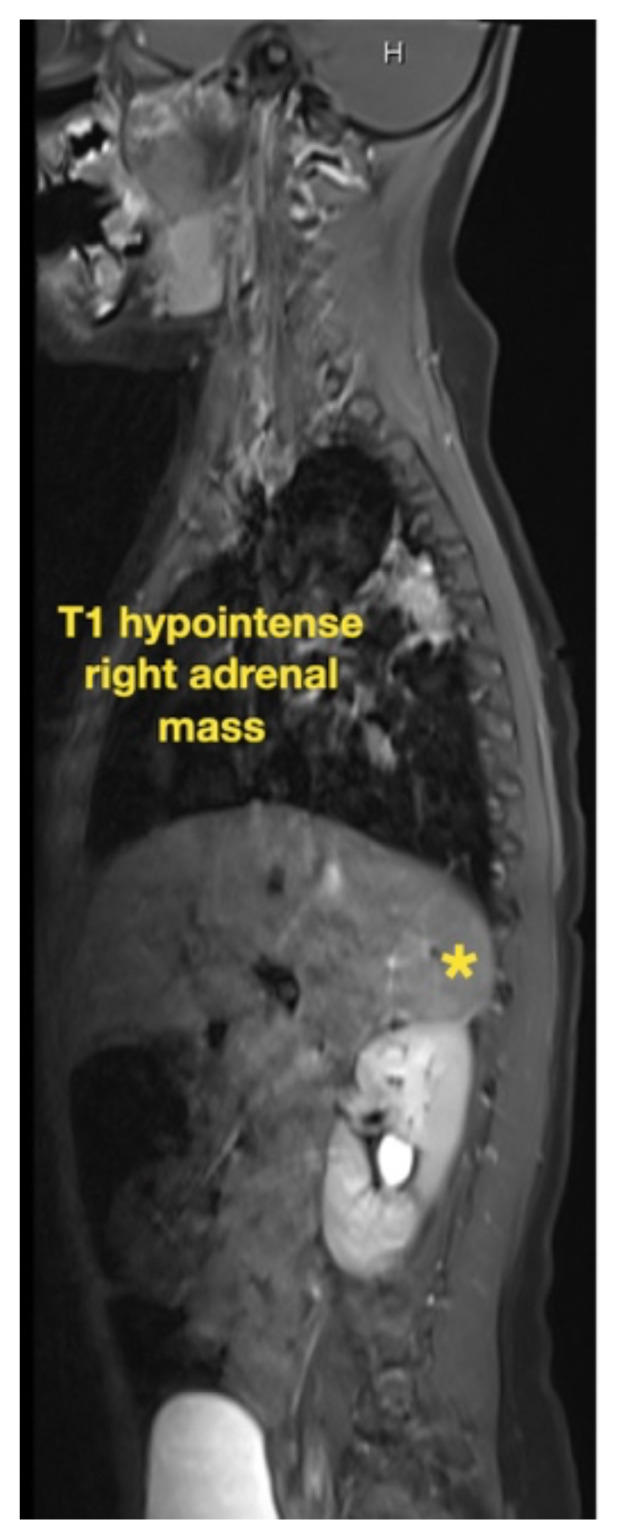


**Figure f2-jetem-7-1-v26:**
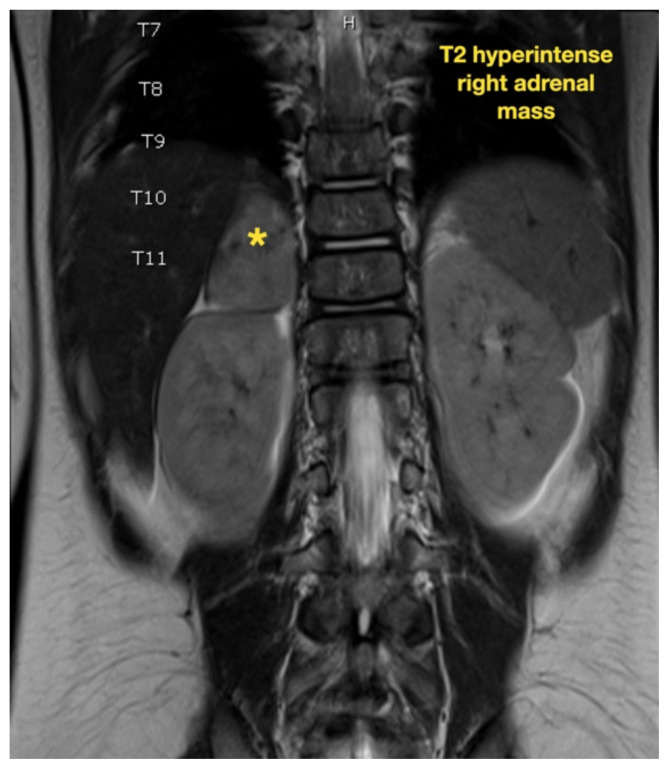


**Figure f3-jetem-7-1-v26:**
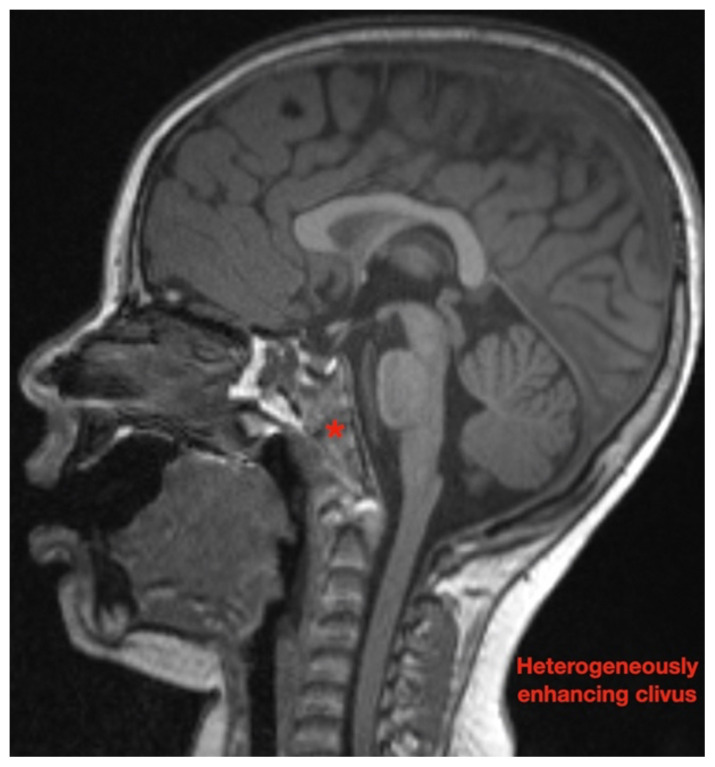


**Figure f4-jetem-7-1-v26:**
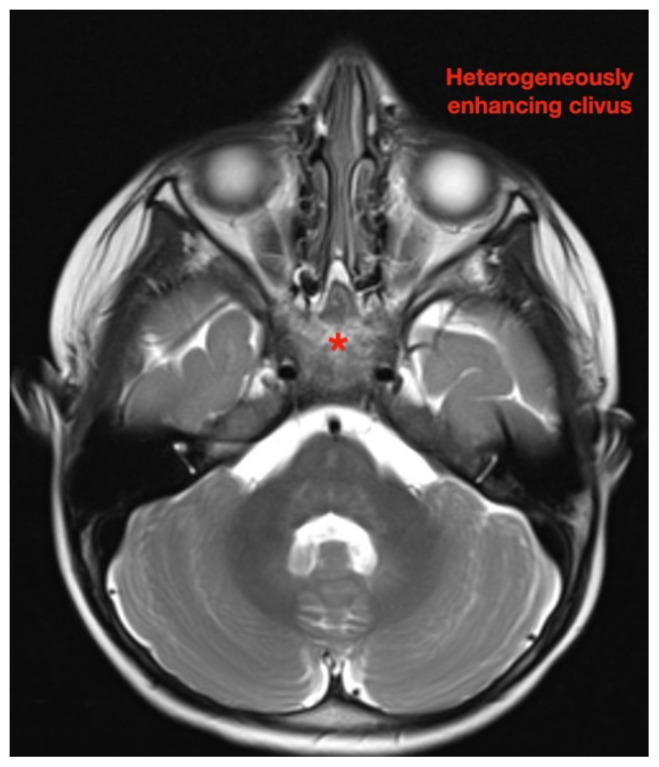


**Figure f5-jetem-7-1-v26:**
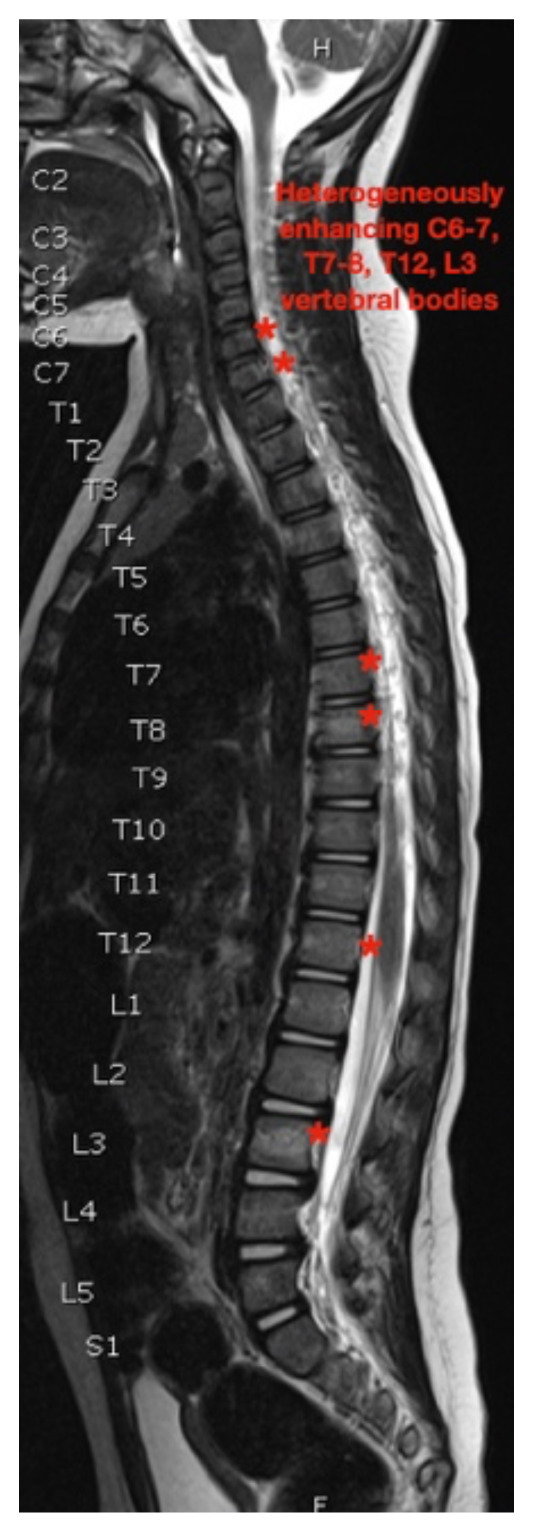


## Brief introduction

Neuroblastoma is the third most common childhood malignancy, trailing leukemia and central nervous system (CNS) tumors, and accounts for 15 percent of childhood cancer mortality.^1^ Peak incidence occurs between 2 to 3 years of age, with a myriad of clinical presentations.^1^,^2^ Bony metastatic disease affects more than half of patients.^3^ Neurologic, ocular, dermatologic, gastrointestinal, and even autoimmune manifestations of the malignancy may mimic other conditions leading to misdiagnosis and a delay in initiation of therapy.^3^ This case report demonstrates an unusual presentation of acute bulbar weakness and ataxia in a toddler presenting to the emergency department (ED), who upon diagnostic evaluation was found to have metastatic neuroblastoma complicated by a suspected paraneoplastic neurologic syndrome. The featured MR imaging illustrates the extent of disease, which in combination with oncologic markers and biopsied pathology, led to the patient’s diagnosis. This case highlights key clinical and imaging findings of neuroblastoma and reviews diagnostic and therapeutic features pertinent to paraneoplastic syndrome.

## Presenting concerns and clinical findings

A 3-year-old previously healthy girl with seven days of recent cough and congestion presented to the ED for 5 days of worsening bilateral ptosis, drooling, choking on liquids, slurring of speech, rightward head tilt and unsteady gait. The symptoms were present constantly throughout the day. On presentation, her parents described her as lethargic, noting a blank stare and sharp reduction in verbal expression. She had no fever, night sweats or weight loss and no preceding trauma. Parents reported no seizure, abnormal eye movements, vision change, difficulty breathing, apparent back pain, or urinary retention. There was no exposure to honey, home-canned foods, or construction sites.

On exam, she was afebrile with normal vital signs, right torticollis, bilateral ptosis and no facial asymmetry or active drooling. There was no meningismus or ophthalmoplegia; pupils were equal and reactive to light with no nystagmus; no respiratory distress; normal muscular bulk and tone; sensation to light touch intact throughout with no sensory level identified; strength 4/5 in upper and lower extremities bilaterally; reflexes 1+ in patellar, biceps and Achilles regions; dysmetric finger-to-nose bilaterally; ambulation with wide-based gait.

Lab work obtained in the ED showed a normal complete blood count and differential with no malignant cells; non-elevated inflammatory markers (ie, erythrocyte sedimentation rate, ferritin, and C-reactive protein); no electrolyte or liver enzyme abnormality; normal thyroid stimulating hormone and free thyroxine; negative urine toxicology and urinalysis; and normal venous blood gas. The patient was found to have parainfluenza 3 virus on comprehensive respiratory polymerase chain reaction (PCR) viral panel testing. A CT head without contrast showed no abnormality, and CT soft tissue neck with contrast showed no significant inflammatory change, abscess or mass lesion. Cerebrospinal fluid from lumbar puncture was unremarkable.

## Significant findings

While still in the ED, MRI with and without gadolinium contrast of the brain, orbits, and cervical, thoracic and lumbar spine were obtained to evaluate for possible CNS lesions including encephalitis, myelitis, or demyelination. Imaging, however, demonstrated multiple unexpected findings: a T1 hypointense, T2 hyperintense and heterogeneously enhancing right adrenal mass measuring 2.7 × 2.1 × 3 cm (yellow asterisk) along with heterogenous enhancement at the clivus, C6, C7, T7, T8, T12, and L3 vertebral bodies (red asterisks). There were otherwise no significant intracranial signal or structural abnormalities and normal orbits.

## Patient course

A neurology specialist consult was placed from the ED and the patient was admitted to the pediatric intensive care unit (PICU) for acute monitoring of bulbar deficits, additional diagnostic workup, and sub-specialist evaluation. The table below summarizes the most relevant differential diagnoses considered along with pertinent clinical, laboratory, and MRI findings. However, considering the patient’s history, exam (ie, hyporeflexia, bilateral ptosis, ataxia) and radiologic findings, she was believed to have possible direct compression of cranial nerves by suspected metastases and/or antibody-mediated paraneoplastic process due to neuroblastoma.

**Table:** 

Diagnosis	Pathophysiology & Clinical Findings	Lab Findings	MRI Brain/Spine w Contrast Findings
Myasthenia Gravis	Neuromuscular junction disorder caused by auto-antibodies to acetylcholine receptors (AChR). Waxing and waning fatigable weakness, ptosis, diplopia.	AChR, muscle-specific kinase (MuSK), or - related low density lipoprotein receptor protein 4 (LRP4) serum auto-antibodies	None
Acute Flaccid Myelitis (AFM)	Post-infectious immune-mediated with temporal spike in late summer/fall; acute asymmetric flaccid limb weakness with diminished reflexes, often accompanied by cranial nerve dysfunction.	CSF pleocytosis	Lesions in grey matter of spinal cord.
Acute disseminated encephalomy elitis (ADEM)	Post-infectious immune-mediated; fever, headache, and encephalopathy with neurologic findings (eg, speech abnormality, ataxia, spinal cord dysfunction, cranial nerve abnormality, upper motor neuron signs).	CSF pleocytosis with possible elevated protein	Lesions in deep and subcortical white matter of brain with periventricular sparing. Extensive transverse myelitis of spinal cord.
Miller Fischer Syndrome	Post-infectious immune-mediated; acute to subacute progressive and symmetric muscle weakness with diminished reflexes; ophthalmoplegia with ataxia.	Elevated CSF protein with normal WBC count (albumin-cytologic dissociation)	Enhancement of spinal nerve roots and/or cauda equina. Cranial nerve roots may also show enhancement.

Neuroblastoma was confirmed on specialized labs demonstrating elevated urine homovanillic acid (HMA) and vanillylmandelic acid (VMA) levels roughly twice the upper limit of normal and a right adrenal mass biopsy demonstrating small round blue cell tumor with scattered stromal spindle cell components. Myasthenia gravis antibody panel and CSF oligoclonal bands returned negative.

Because paraneoplastic syndrome is an antibody-mediated process, immune modulating therapy was initiated with a glucocorticoid (methylprednisolone 0.7 mg/kg daily for 3 days) and plasmapheresis (5 days). The latter involves drawing blood via a large bore catheter which is then centrifuged in a pheresis machine to draw off plasma (which contains autoantibodies) and replaced with 5% albumin. Following this, the patient continued to experience dysphagia, ptosis, and ataxia with no deterioration of clinical status. Chemotherapy was initiated and the patient experienced gradual improvement and is now in remission.

## Discussion

Neuroblastoma accounts for 8 percent of childhood cancers and is the most common extracranial solid tumor in children.^4^ It is most common in infants, and the prognosis worsens with increasing age at diagnosis.^5^ Neuroblastomas may arise in any location along the sympathetic nervous system due to origination from embryonic neural crest cells.^6^ The most common primary sites are the adrenal gland (40%), followed by the abdomen (25%), thoracic (15%), cervical (5%), and pelvic sympathetic ganglia (5%).^7^,^8^ Metastatic spread occurs most commonly to the liver and lymph nodes, but may also spread intracranially.^9^ Patients may present with a wide range of clinical features depending on body site(s) affected, with more characteristic signs and symptoms including abdominal mass, periorbital ecchymoses (“raccoon eyes”), constitutional symptoms, or bone pain.

Patients may also manifest with abdominal pain, constipation, or back pain with weakness (from vertebral or spinal cord compression).^10^ Up to 16 percent of patients may demonstrate ocular signs, including direct orbital involvement, opsoclonus, and Horner syndrome (from cervical or cervico-mediastinal mass).^11^ This case depicts challenges in early recognition of neuroblastoma. Bilateral ptosis and muscle weakness as initial presenting signs of neuroblastoma are rarely reported in the literature.^12^ Such a constellation of symptoms may be more readily attributable to neurologic diagnoses such as those featured in the table above, or even brainstem tumors.

Imaging findings range just as widely as clinical presentations. While CT, MRI and bone scintigraphy are used for disease staging, MRI is more sensitive in detecting bony involvement, including assessment of intra-foraminal extension of tumor and spinal cord compression.^6^ This is especially relevant because skeletal metastases occur in up to 60 percent of cases.^3^ Long bones and vertebrae may demonstrate pathologic fractures, osteolysis, periosteal reaction, or radiolucent streaks.^13^ Cranial bones may appear heterogenous and cranial suture lines may even widen due to adjacent dural metastases.^13^ MRI is not only essential in diagnosis and staging but also in defining the relation of the tumor to vital structures, tumor resectability, post-excisional residual disease, treatment efficacy, and disease surveillance.^14^

Opsoclonus-myoclonus is a type of paraneoplastic syndrome that occurs in 1 to 3 percent of pediatric patients with neuroblastoma and is believed to be autoimmune in nature.^15^ The classic syndrome of rapid, dancing eye movements and rhythmic jerking of limbs and/or trunk may be accompanied by or preceded by ataxia.^15^ It is not entirely clear in this patient’s case if ataxia was an early presenting sign of paraneoplastic syndrome, metastatic compression of intracranial nerves, or due to pain from vertebral metastatic spread.

In summary, neuroblastomas are uniquely heterogeneous tumors, presenting in a variety of locations, provoking a spectrum of neurologic symptoms, and behaving widely, from spontaneous regression to aggressive life-threatening metastatic disease. There are little known risk factors, making a detailed clinical exam, index of suspicion, and appropriate imaging critical to a timely diagnosis. For all these reasons, precise delineation of the extent of neuroblastoma and individualized strategy for patient therapy are paramount.

## Supplementary Information










