# Supplementary figures and images for: An Unusual Case Report of a Toddler with Metastatic Neuroblastoma Mimicking Myasthenia Gravis
Source: J Educ Teach Emerg Med. 2022 Jan 15;7(1):V26–30. doi: 10.21980/J8G35V (PMC10358866; doi:10.21980/J8G35V)

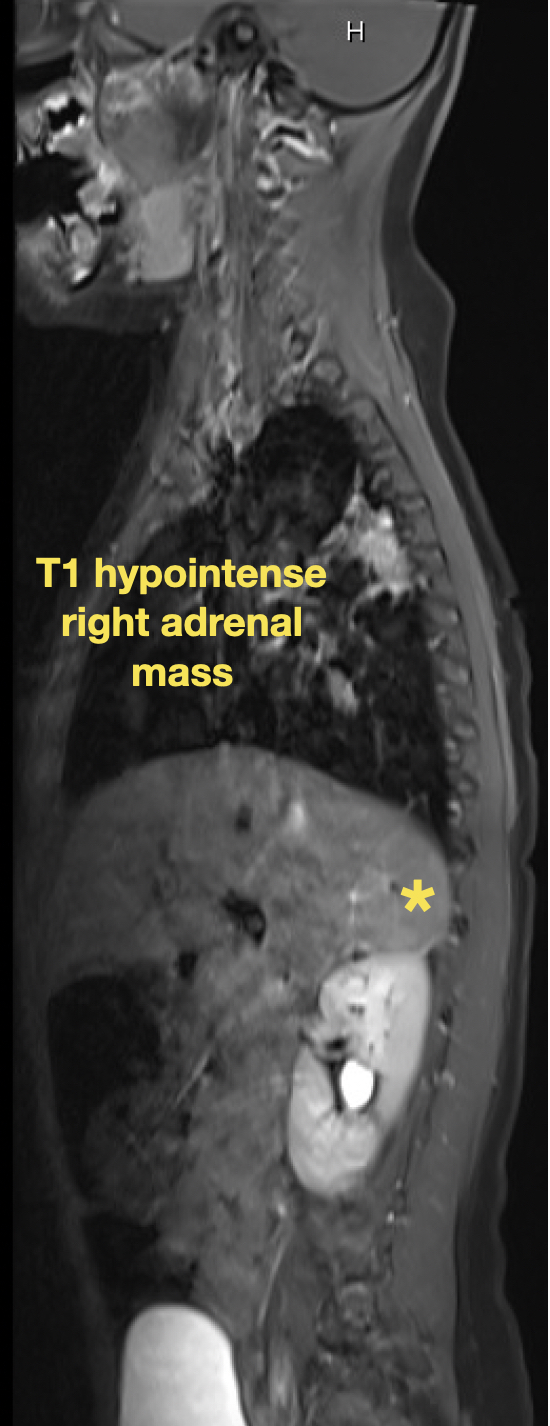

Supplement: Supplementary file 1 [file JETem-7-1-V26-supp1.jpg]

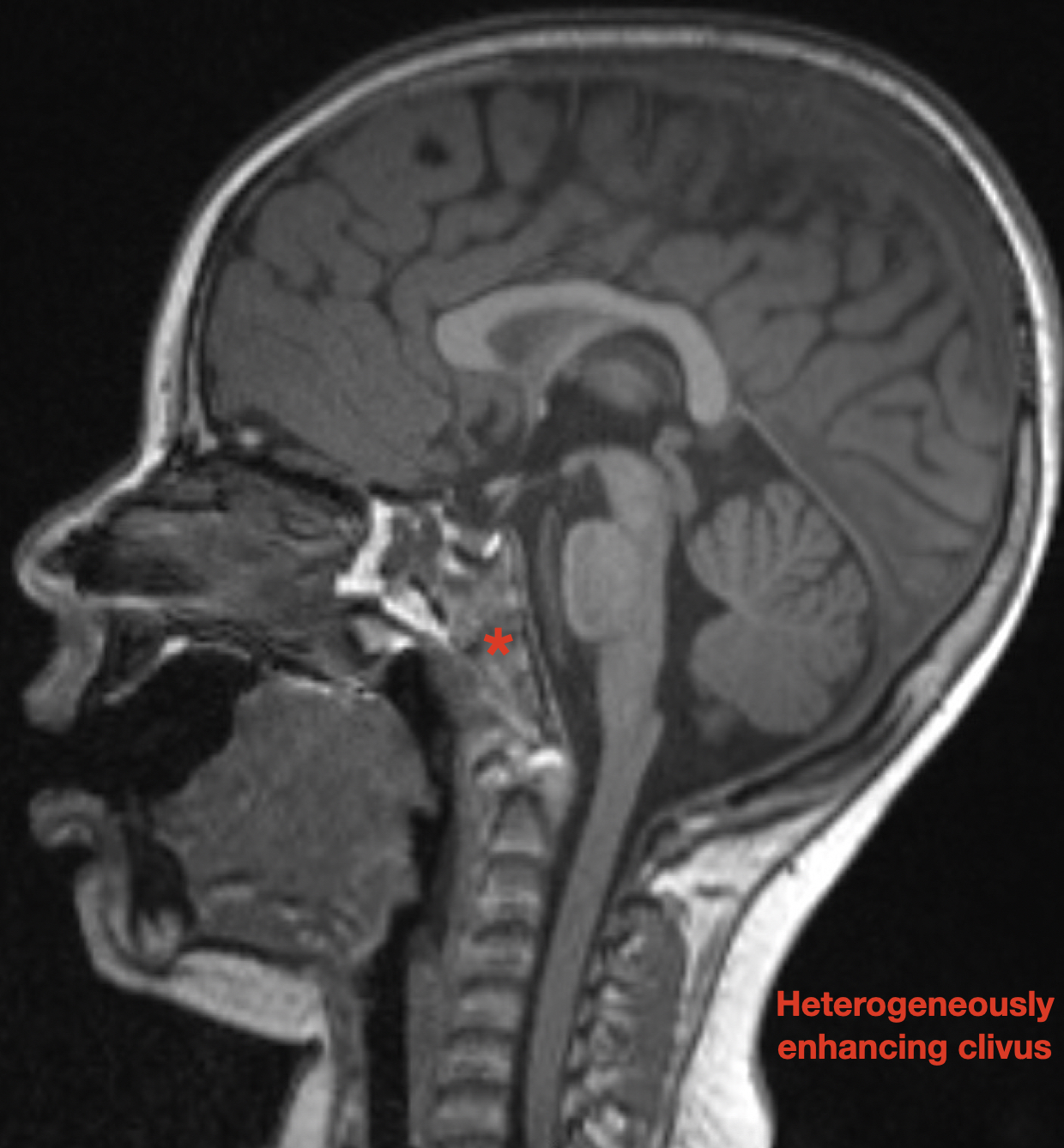

Supplement: Supplementary file 2 [file JETem-7-1-V26-supp2.jpg]

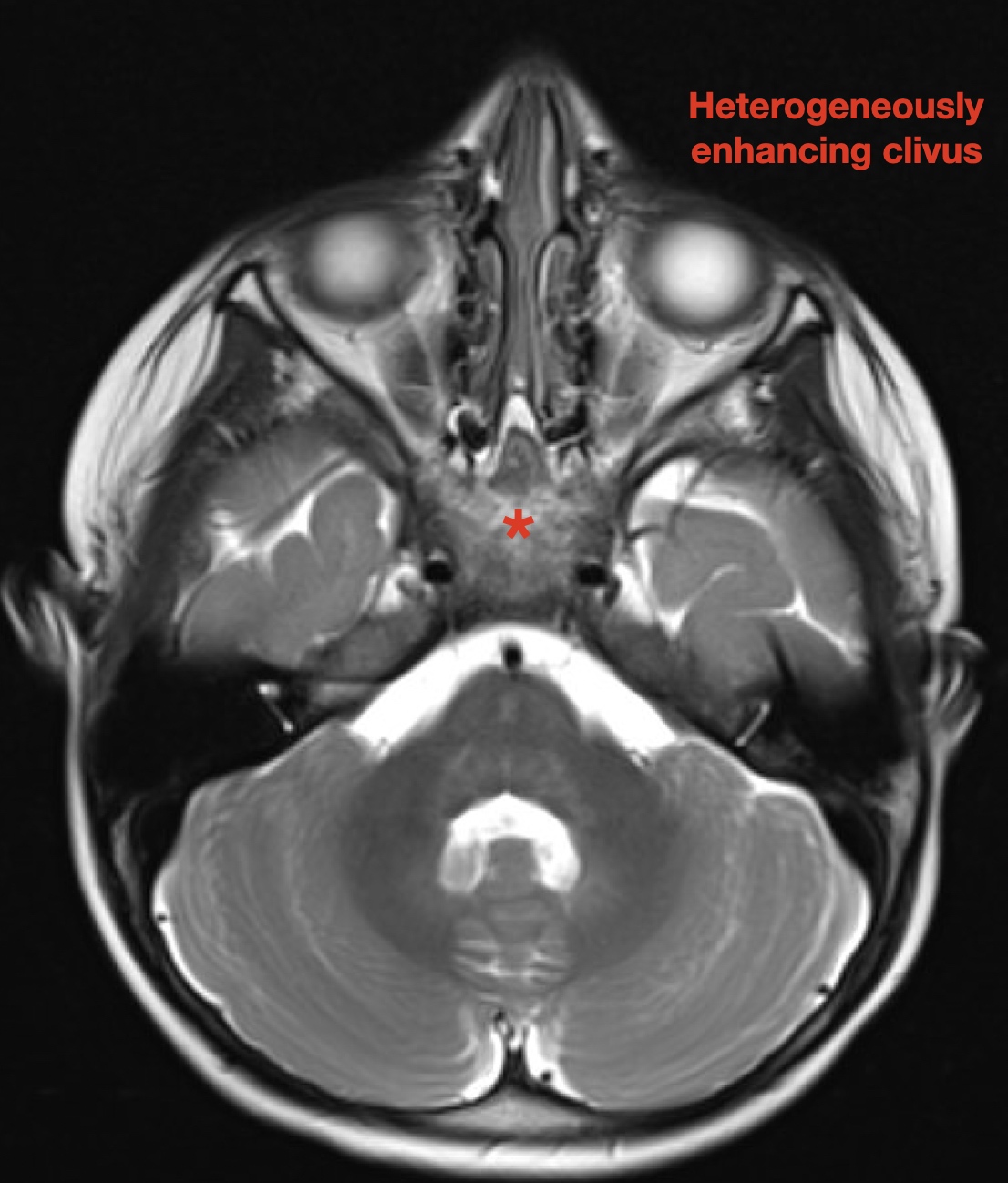

Supplement: Supplementary file 3 [file JETem-7-1-V26-supp3.jpg]

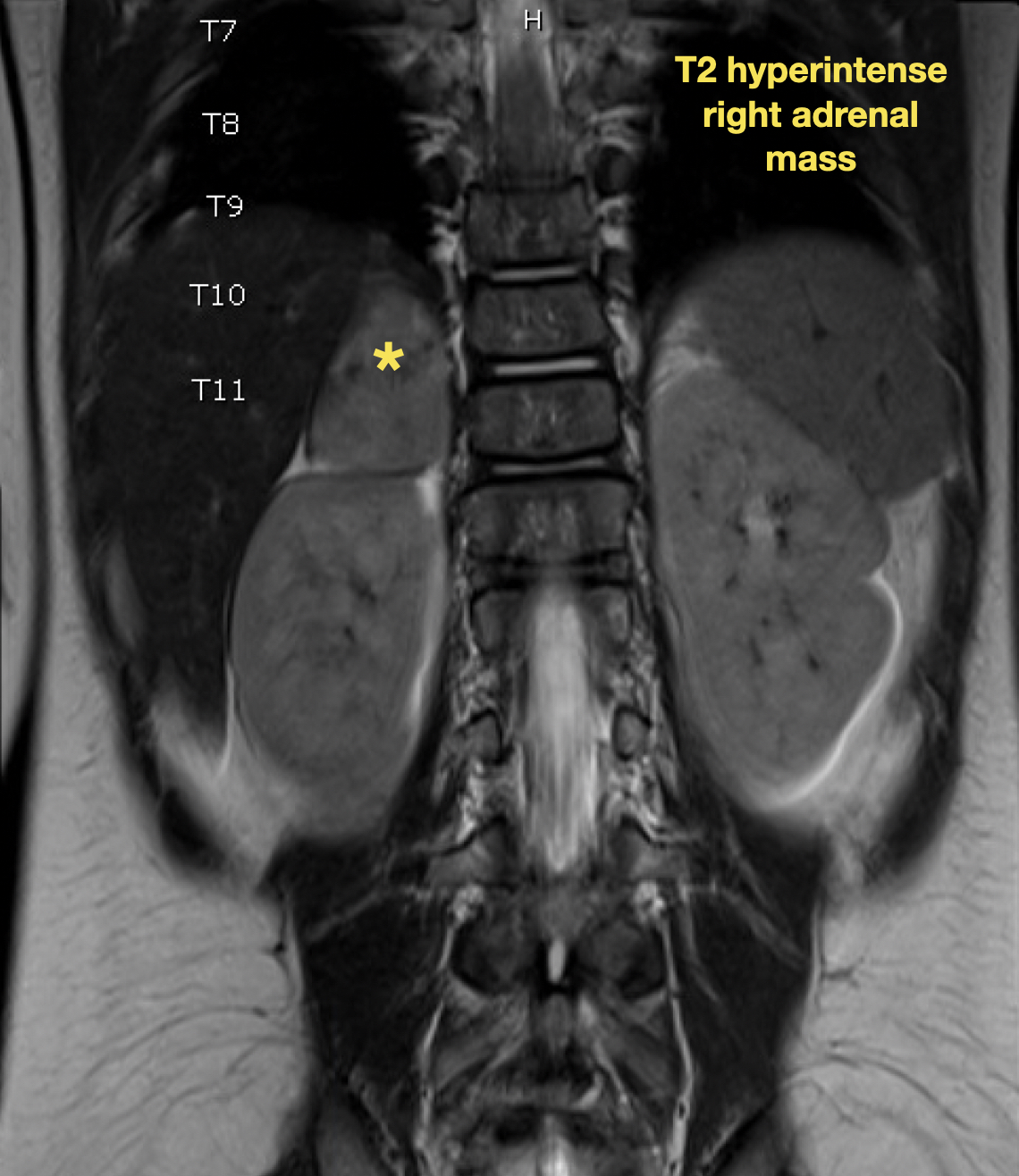

Supplement: Supplementary file 4 [file JETem-7-1-V26-supp4.jpg]

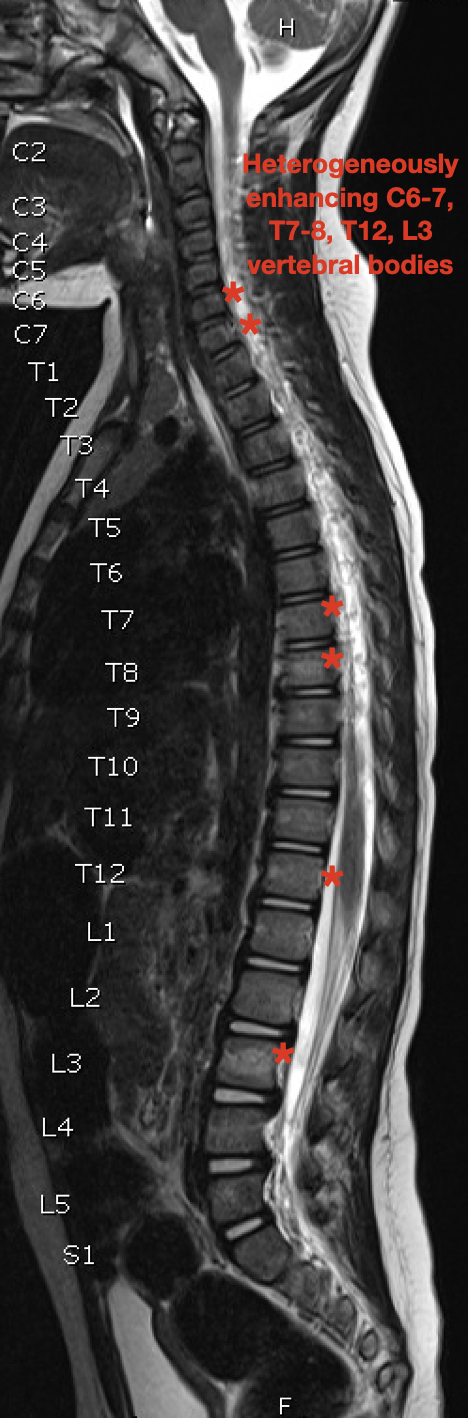

Supplement: Supplementary file 5 [file JETem-7-1-V26-supp5.jpg.jpg]
